# Optimization of Immunoglobulin Substitution Therapy by a Stochastic Immune Response Model

**DOI:** 10.1371/journal.pone.0005685

**Published:** 2009-05-28

**Authors:** Marc Thilo Figge

**Affiliations:** Frankfurt Institute for Advanced Studies (FIAS), Goethe University Frankfurt, Frankfurt am Main, Germany; New York University School of Medicine, United States of America

## Abstract

**Background:**

The immune system is a complex adaptive system of cells and molecules that are interwoven in a highly organized communication network. Primary immune deficiencies are disorders in which essential parts of the immune system are absent or do not function according to plan. X-linked agammaglobulinemia is a B-lymphocyte maturation disorder in which the production of immunoglobulin is prohibited by a genetic defect. Patients have to be put on life-long immunoglobulin substitution therapy in order to prevent recurrent and persistent opportunistic infections.

**Methodology:**

We formulate an immune response model in terms of stochastic differential equations and perform a systematic analysis of empirical therapy protocols that differ in the treatment frequency. The model accounts for the immunoglobulin reduction by natural degradation and by antigenic consumption, as well as for the periodic immunoglobulin replenishment that gives rise to an inhomogeneous distribution of immunoglobulin specificities in the shape space. Results are obtained from computer simulations and from analytical calculations within the framework of the Fokker-Planck formalism, which enables us to derive closed expressions for undetermined model parameters such as the infection clearance rate.

**Conclusions:**

We find that the critical value of the clearance rate, below which a chronic infection develops, is strongly dependent on the strength of fluctuations in the administered immunoglobulin dose per treatment and is an increasing function of the treatment frequency. The comparative analysis of therapy protocols with regard to the treatment frequency yields quantitative predictions of therapeutic relevance, where the choice of the optimal treatment frequency reveals a conflict of competing interests: In order to diminish immunomodulatory effects and to make good economic sense, therapeutic immunoglobulin levels should be kept close to physiological levels, implying high treatment frequencies. However, clearing infections without additional medication is more reliably achieved by substitution therapies with low treatment frequencies. Our immune response model predicts that the compromise solution of immunoglobulin substitution therapy has a treatment frequency in the range from one infusion per week to one infusion per two weeks.

## Introduction

Adaptive immunity implies immune responses against pathogenic challenges that are antigen-specific and that are memorized by the immune system. On encounter of antigen, B-lymphocytes are stimulated to differentiate into plasma cells which produce large amounts of immunoglobulin. These proteins are specific for those antigens that stimulate their production and play a key role in adaptive immunity: Immunoglobulin fights off bacterial infections by the specific recognition of the invading pathogens, the neutralization of their harmful effects, and their opsonization for phagocytosis [1], [2].

In order to specifically bind to the vast amount of different antigens, the molecular structure of immunoglobulin contains a hypervariable region. This region is generated by random combinations of gene segments that encode a large variety of antigen binding sites and that give rise to a highly diverse repertoire of immunoglobulin. The immunoglobulin binding affinity for an encountered antigen is dynamically optimized in the process of affinity maturation that takes place in germinal centers. Germinal centers are follicular structures in lymphoid organs where B-lymphocytes undergo the process of somatic hypermutation with regard to the immunoglobulin hypervariable region [3]–[5]. This is followed by the complex process of B-lymphocyte selection for high-affinity immunoglobulin, which we only start to unravel today [6]. Successfully selected B-lymphocytes either differentiate into plasma cells or into long-lived memory cells. The latter give rise to faster and stronger immune responses on second encounter of the same antigen. In this way the highly diverse immunoglobulin repertoire is dynamically adapted to the host's current antigenic environment.

In humans, five different immunoglobulin isotypes are distinguished that differ in their biological and functional properties [2]. The most prevalent isotype is immunoglobulin G (IgG), which constitutes about 75% of all serum immunoglobulin and is equally distributed in blood and in tissue. IgG is the only isotype that crosses the human placenta thereby protecting the fetus in utero and providing neonates with passive immunity for the first six months of their life, before the infant's immune system starts to produce its own immunoglobulin. Thus, rather than being present at birth, adaptive immunity is an acquired property of the developing immune system in healthy infants.

Patients with immune deficiencies suffer from recurrent and persistent infections that develop as the result of a compromised immune system [7]. For example, X-linked agammaglobulinemia (XLA) is a primary immune deficiency that is characterized by absent levels of immunoglobulin for all isotypes [8]–[10]. This disease is caused by a mutation of Bruton's tyrosine kinase (Btk gene) on the X chromosome [11], [12] and affects male and homozygous female subjects. The genetic defect prohibits the full maturation of B-lymphocytes such that their vital function in immunoglobulin adaptation and production remains unaccomplished. Recurrent infections in infants starting after the first six months of life, particularly involving extracellular bacteria, are typical phenomena belonging to the XLA diagnosis. Untreated XLA patients are prone to develop severe and life-threatening infections, however, this risk is significantly lowered by IgG substitution therapy. The life-long, exogenously induced passive immunity compensates the absence of adaptive immunity as mediated by the B-lymphocytes and enables XLA patients to live a fairly normal life.

Under IgG substitution therapy, a fixed dose of IgG is periodically administered by infusion, such that the serum IgG level always remains above a trough level [13]–[16]. The pool of immunoglobulin is extracted from the blood products of more than 10^3^ human donors in order to reach up to 10^9^ different immunoglobulin specificities [13]. Nowadays, IgG substitution treatments are performed by intravenous (IV) or subcutaneous (SC) infusion that both have assets and drawbacks [15], [17]–[19]. For example, IV infusion allows the administration of large IgG amounts per treatment such that low treatment frequencies down to one treatment per four weeks can be achieved. However, this IV treatment protocol gives rise to high serum IgG peak levels that significantly exceed the physiological range, but are required in order to ensure acceptable serum IgG trough levels after four weeks. The drawback of SC infusion is associated with the limited amount of IgG that can be administered per treatment and that requires high treatment frequencies up to two times per week. However, this also infers serum IgG peak levels that remain close to the physiological range. Associated with this issue is the observation that adverse effects are much less reported for SC compared to IV infusion of IgG [19]. A practical advantage of SC over IV infusion is that this treatment can be autonomously performed by the patients and does not require medical supervision.

Besides immune deficiencies a number of autoimmune and systemic inflammation diseases are as well treated by IgG substitution therapy, even though it is not yet understood for all these diseases how IgG exerts its therapeutic impact [13], [14]. In general, the demand for therapeutic use of IgG is increasing at an annual rate of 5% in Europe and 11% in the US, prophesying that the current worldwide IgG shortage will pose a serious problem in the future [20], [21]. To optimize IgG consumption the appropriate dosage window of treatment, which is commonly derived from empirical trial-and-error methods, should be more precisely determined by all means. Using a mathematical model approach, we perform a comparative study of IgG substitution therapies for XLA patients in order to identify the range of optimal treatment frequencies. Characteristic features such as the serum IgG peak level, the dose per treatment, and the impact of fluctuations in the administered IgG dose are studied. Moreover, we consider the effect of bacterial infections and calculate the condition for the development of chronic infections under different IgG substitution therapies. The range of optimal treatment frequencies is then identified from the requirement that IgG levels should be kept close to physiological levels and are still effective at clearing infections. To our knowledge, we present the first immune response model for infections under IgG substitution therapy with a combined analysis of computer simulations and analytical calculations that permits to make quantitative predictions of therapeutic relevance.

## Methods

### Stochastic Immune Response Model

**Figure 1:**
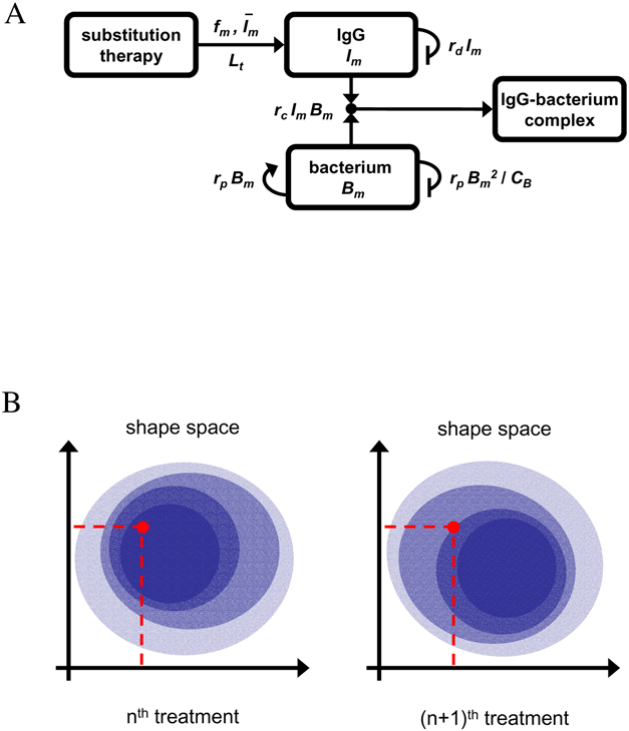
Schematic representation of the stochastic immune response model under IgG substitution therapy. A: The serum IgG trough level 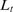 is maintained by substitution therapy with treatment frequency 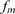 and IgG dose 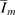 per treatment (infusion process: →). The natural degradation of IgG occurs with rate 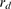 (self-inhibiting process: ⊣) and the binding of IgG to bacteria occurs with clearance rate 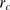 (binding process: →•←), giving rise to IgG-bacterium complexes that are removed from the system. The bacteria population obeys a logistic growth dynamics that is characterized by the proliferation rate 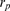 (self-activating process: →) and the carrying capacity 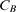 (self-inhibiting process: ⊣). B: The shape space is an abstract high-dimensional space, where essential features of IgG binding regions with respect to the considered bacteria species are represented as points. For reasons of clarity, a two-dimensional shape space is depicted where the two axis represent independent features of IgG binding, such as charge and size of the binding site. The inhomogeneous IgG shape space distribution under substitution therapy with pooled IgG (bluish area) is represented by the color intensity (arbitrary units). Even if the total amount of administered IgG per treatment is exactly the same, with respect to a specific bacterium, i.e. a specific shape space area (red point), fluctuations occur from treatment to treatment.

The stochastic immune response model for infections under IgG substitution therapy accounts for two aspects: (i) the serum IgG reduction by natural degradation or antigenic consumption, and (ii) the periodic IgG replenishment that may be subjected to fluctuations in the administered IgG dose. The model is schematically depicted in Figure 1A and is translated into a set of coupled differential equations. Since we are interested in describing serum IgG levels, this approach, in which spatial inhomogeneities are neglected, is justified by the fact that molecules in the blood stream are thoroughly mixed and quickly circulate on the time scale of 25 seconds [22]. Throughout this paper we measure concentrations of quantities relative to the blood volume in units of gram per liter.

The time-dependent serum IgG concentration, 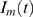, obeys the differential equation
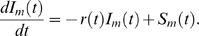
(1)The source term 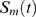 represents the IgG substitution therapy, where the subscript 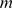 refers to the treatment frequency 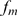 of the therapy protocol. Since the longest time interval between subsequent IV infusion treatments equals four weeks [14], [15], we set 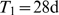 and 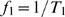. In order to compare therapy protocols with different treatment frequencies, we consider therapy protocols with multiples of this frequency:
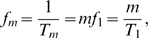
(2)where 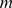 is a positive integer number. The IgG reduction in Eq. (1) is represented by the rate 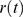, which consists of two contributions:
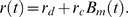
(3)Here, 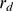 is the natural IgG degradation rate while the antigenic consumption depends on the current size of the bacteria population 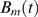 and the clearance rate 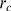. Note that the clearance rate is a reaction rate with unit (concentration×time)^−1^.

A large variety of sigmoidal growth models exists, describing the time-dependent growth of specific microbes under various nutrient conditions [23]–[25]. In the present context, we do not aim at modeling specific types of bacteria and apply a logistic growth model, which is known to capture the generic features of nutrient-limited bacterial growth beyond the lag-phase [25]. In the presence of the immune response the time-dependence of the bacteria population is determined by the differential equation:
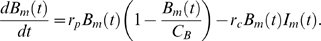
(4)Bacteria proliferate with rate 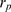 to the nutrient-limited population size with carrying capacity 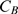 in the absence of an immune response. Note that the subscript 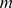 indicates that the time-evolution of 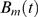 is affected through 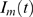 by the frequency 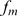 of the therapy protocol.

The IgG substitution therapy is incorporated by the source term
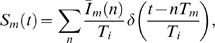
(5)where 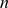 labels treatments of duration 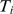 at time points 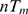, and
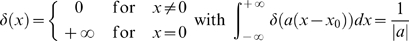
(6)is the Dirac distribution. The appearance of the treatment duration 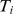 in Eq. (5) is only artificial, since the Dirac distribution implies that infusion treatments are modeled as instantaneous processes. This simplification is justified by the fact that 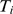 is of the order of hours, whereas the time between subsequent treatments is of the order of several days, 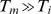, and we are ultimately interested in the time-dependence of 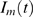 and 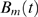 at the time scale of weeks, 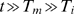.

We account for fluctuations in the administered IgG dose per treatment,
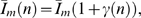
(7)where 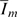 is the average administered IgG dose per treatment and 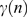 is a Gaussian distributed random variable referring to the 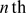 treatment. The ensemble average 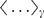 yields the mean value
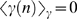
(8)and the correlation function
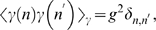
(9)where
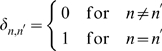
(10)is the Kronecker delta. The dimensionless constant 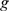 denotes the standard deviation of the Gaussian distribution and is a measure for the strength of the fluctuations. Thus, Eq. (7) enters the source term Eq. (5) and represents fluctuations in the total amount of administered IgG per treatment. As a consequence, Eq. (1) for 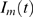 represents a stochastic differential equation and the stochasticity of 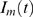 enters Eq. (4) for 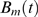 via the immune-response term.

In the population model, we do not explicitly resolve IgG specificities and different species of bacteria. A detailed description could be realized within the shape space concept [26]. The shape space is an abstract high-dimensional space, where essential features of IgG binding regions with respect to the considered bacteria species are represented as points. In this spirit, points in the shape space become occupied by the pooled IgG that is administered in each treatment. However, the occupation of shape space points does not necessarily occur in a homogenous fashion. As is schematically shown in Figure 1B, even for the same total amount of administered IgG, the amount of IgG that is available to specific shape space areas can be fluctuating from treatment to treatment. Since the immune-response term in Eq. (4) is the product of 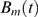 and 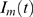, the fluctuations Eq. (7) in the administered IgG dose per treatment can be viewed as an effective description of shape space inhomogeneities within the population model.

It is straightforward to compute the time-evolution of the serum IgG concentration and the bacteria concentration by numerical integration of Eqs. (1) and (4). Moreover, the advantage of our model is that it permits to analytically calculate closed expressions for undetermined quantities such as the clearance rate.

### Computer Simulations

We perform simulations of the model Eqs. (1) and (4) using a self-written algorithm that is based on the fourth-order accurate Runge-Kutta method [27]. In all simulations the time step of integration is set to the typical infusion duration per treatment, 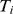, which is estimated to be two hours. The model parameters are summarized in Table 1 and we checked that our numerical results are robust against small variations in the parameter values.

**Table 1:** Overview of model parameters.

parameter description	parameter value	comment
IgG trough level	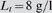	Refs. [13], [15], [16]
IgG treatment duration	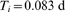	simulation time step
IgG treatment frequency	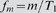	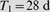 , 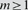
IgG dose per treatment	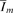	adjusted to maintain 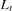
IgG dose fluctuation strength	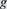	varied
IgG degradation rate	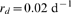	Refs. [29], [30]
bacteria clearance rate	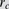	varied
bacteria proliferation rate	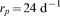	generic value
bacteria carrying capacity	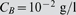	generic value
time point of infection	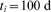	generic value
initial bacteria population size	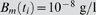	generic value
minimal bacteria population size	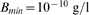	estimated

### Analytical Calculations

#### Substitution Therapies in the Absence of Infections

In the absence of infections, the model reduces to Eq. (1) with 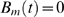 and the source term Eq. (5) for substitution therapy with frequency Eq. (2). It can be easily verified by differentiation that the model is solved by the serum IgG concentration
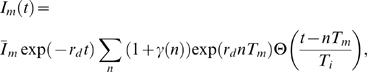
(11)where the step function,
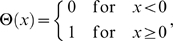
(12)ensures that only treatments 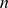 with 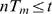 contribute.

After 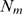 elapsed treatments, the next treatment takes place at 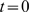 and the time-evolution of 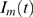 during the interval 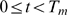 is given by
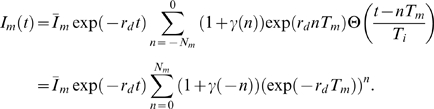
(13)The number of treatments scales as 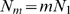, such that the total elapsed time, 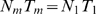, is equal for all 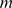. In the equilibrated system the number of elapsed treatments 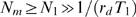, implying that Eq. (13) reduces to
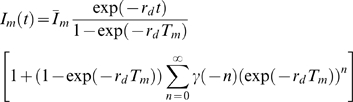
(14)where we used the limiting value of the infinite geometric series. The ensemble average of Eq. (14) yields
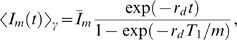
(15)which is identical to 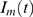 in the absence of fluctuations. Furthermore, the ensemble average of 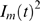 yields
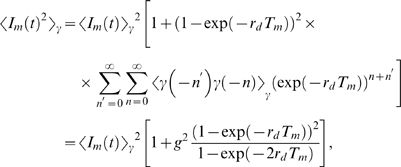
(16)where we used Eq. (9) and the limiting value of the infinite geometric series. The expression Eq. (16) gives rise to the relative variance
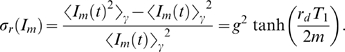
(17)


### Substitution Therapies in the Presence of Infections

In the presence of fluctuations, we solve Eq. (4) with
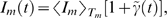
(18)where we account for fluctuations around the time-averaged serum IgG concentration 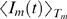 according to Eq. (7). Thus, modulations of 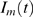 on time scales smaller than 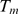 are averaged out while the fluctuations in the administered IgG dose at time intervals 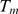 are represented by a continuous function of Gaussian distributed random numbers with
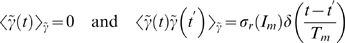
(19)in terms of the relative variance Eq. (17). It is convenient to perform the variable transformation
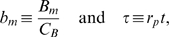
(20)which reduces Eq. (4) to
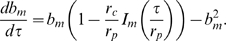
(21)This equation can be readily solved by computer simulations for different random realizations of fluctuations in order to compute the average size of the bacteria population as a function of time. Alternatively, we derive and solve the Fokker-Planck equation that corresponds to Eq. (21) in order to obtain the probability distribution 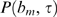 for the time-dependent bacteria population size.

In general, the probability that the bacteria population has size 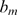 at time 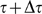 is given by
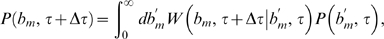
(22)where 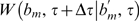 denotes the transition probability from 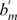 to 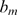 during the time interval 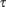 to 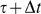. For the Gaussian fluctuations Eq. (19), it can be shown that the transition probability reduces to the expression [28]:
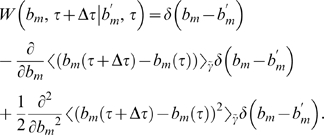
(23)Inserting Eq. (23) into Eq. (22) and taking the limit 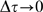 yields the Fokker-Planck equation:
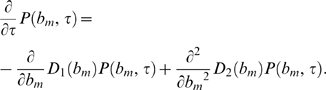
(24)This equation describes the time-evolution of the probability distribution 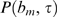 with initial bacteria concentration 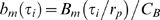 at the time point of infection 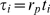:
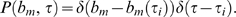
(25)The coefficients in Eq. (24) are defined by
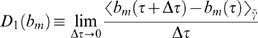
(26)and
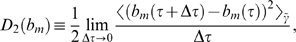
(27)and are derived on the basis of Eq. (21):
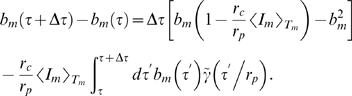
(28)We readily obtain
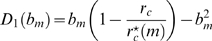
(29)in terms of the critical clearance rate [cf Eq. (74)],
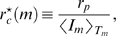
(30)while the only term contributing to Eq. (27),
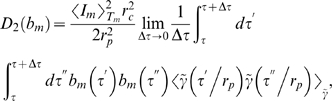
(31)evaluates to
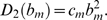
(32)Here, we defined the dimensionless factor
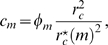
(33)which contains the non-negative quantity 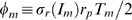.

For an equilibrated system, the probability distribution is obtained from Eq. (24) under the condition
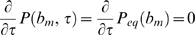
(34)or, equivalently, as the solution of
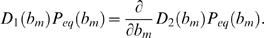
(35)This equation is trivially solved for 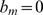. In particular, in the absence of fluctuations 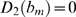 and we readily obtain that 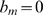 is realized for 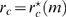.

In the presence of fluctuations, Eq. (35) becomes for 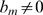:
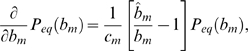
(36)with
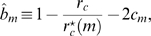
(37)where the distribution has its maximum. The solution of Eq. (36) is given by
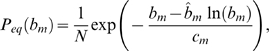
(38)where the normalization constant,
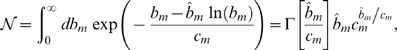
(39)contains the Gamma function, 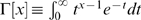, which is valid for 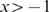.

We are now in the position to calculate the average size of the bacteria population,
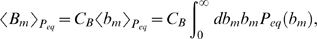
(40)and obtain the result:
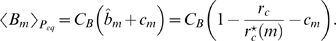
(41) Similarly, averaging the square of the bacteria population yields:
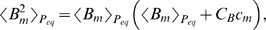
(42)such that the relative variance of the distribution is given by
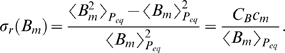
(43)In terms of Eqs. (41)–(43), the probability distribution Eq. (38) can be written in the form
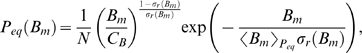
(44)which can be further reduced to the expression Eq. (75).

Since the condition Eq. (34) entails that the time-dependence of 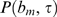 is lost, we estimate the clearance time 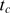 after which the infection is cleared in the presence of fluctuations. Victorious fluctuations can compensate for a reduced time-averaged IgG concentration: 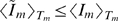. The condition Eq. (30), which is valid in the absence of fluctuations, translates in formal analogy to Eq. (7) into
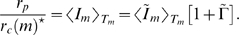
(45)To estimate the number of treatments required for the victorious fluctuation to occur, we average the sum of fluctuations for 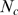 treatments,
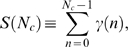
(46)under the constraint that it yields at least the value
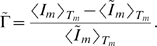
(47)Thus, on average the victorious fluctuation 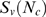 is given by
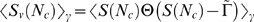
(48)in terms of the step function Eq. (12). The brackets denote the average
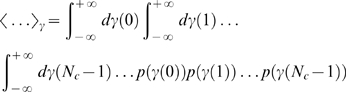
(49)with the Gaussian distribution
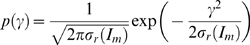
(50)in terms of the variance Eq. (17). Using the integral representation of the step function in the complex plane, we have to solve
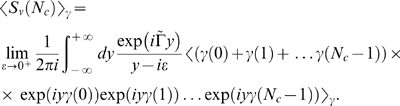
(51)Taking the average yields
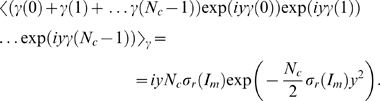
(52)Inserting this expression into Eq. (51) and integrating over 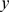, we obtain the result:
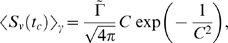
(53)where we introduced
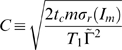
(54)and substituted 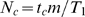.

The condition Eq. (47), according to which the victorious fluctuation compensates for the reduced time-averaged serum IgG concentration, implies 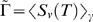. In this case, the numerical solution of Eq. (53) is given by 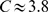, so that Eq. (54) gives rise to the clearance time
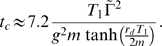
(55)Linearizing the tangens hyperbolicus for 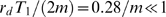 yields the expression Eq. (87).

## Results

### Substitution Therapies in the Absence of Infections

We consider IgG substitution therapies that differ in the treatment frequency in order to compare serum IgG peak levels 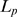 and IgG dosages that are required to maintain the serum IgG trough level 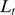. Since it has been reported that XLA patients require a trough level of 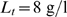 to prevent recurrent bacterial infections [13], [15], [16], we use this value in all calculations. Similarly, we keep the degradation rate 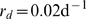 fixed in all calculations. This value is based on the *in vivo* half-lifetime for IgG blood products of about 35 days, as consistently reported by the American Society of Health-System Pharmacists [29] and the Canadian Blood Services [30].

**Figure 2:**
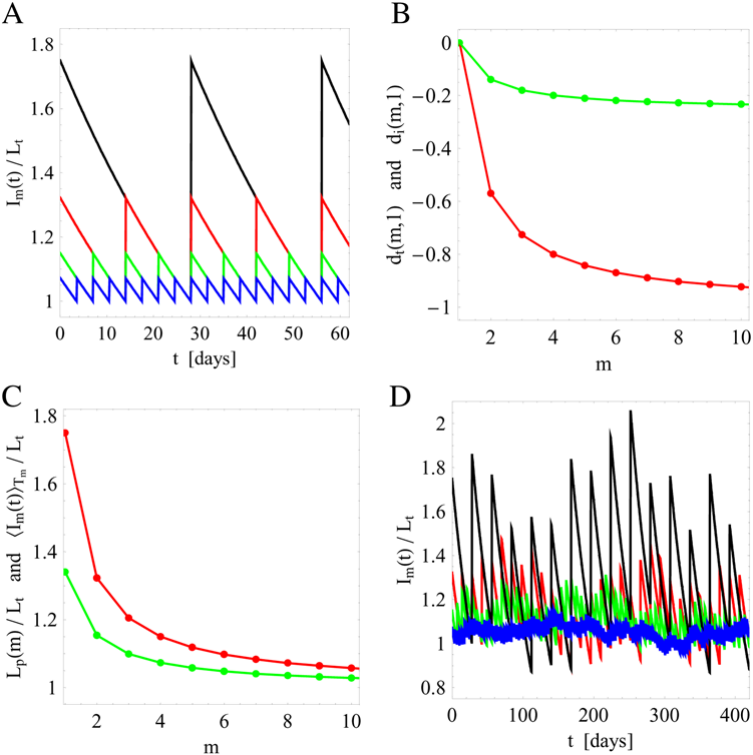
Comparison of serum IgG concentrations for different substitution therapies in the absence of infections. A: Simulation results based on Eq. (1) with 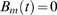 and the source term Eq. (5) for different substitution therapies in the absence of fluctuations (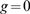). The frequencies of the substitution therapies are: 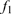 (black line), 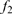 (red line), 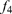 (green line), and 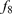 (blue line). B: Analytical calculation of the relative difference in the IgG doses per treatment 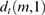 (red line) and time-integrated 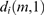 (green line) for different substitution therapies according to Eqs. (59) and (60), respectively. C: Analytical calculation of the serum IgG peak level 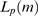 (red line) and the time-averaged dose 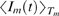 (green line) for different substitution therapies according to Eqs. (61) and (62), respectively. D: The same as in A but in the presence of fluctuations in the administered IgG amount per treatment with fluctuation strength 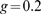.

In the absence of infections and fluctuations in the administered IgG dose, the model reduces to Eq. (1) with 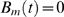 and the source term Eq. (5) with 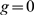 for substitution therapies of frequency 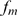. The computed time-evolution of the serum IgG concentration 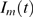 is presented in Figure 2A, where we compare four substitution therapies that differ by the time intervals 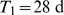, 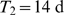, 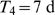, and 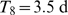 between subsequent treatments. For substitution therapy with frequency 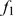 the simulation predicts that a dose of 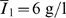 per treatment is required to maintain the serum IgG trough level 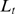. This value is in agreement with the empirical protocol of IV infusion therapy, which prescribes 0.4 g IgG per kg body weight every four weeks [14], [15]. For an adult person with a body weight of 75 kg and a blood volume of 5 liters, this implies 30 g IgG per treatment and equals the administered serum IgG concentration of 6 g/l. For substitution therapies with higher frequencies, the simulations predict IgG doses per treatment of 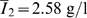 for 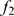, 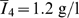 for 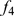, and 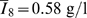 for 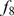. In the case of substitution therapy with treatment frequency 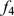 this corresponds to 0.08 g IgG per kg body weight every week and compares reasonably well with extended studies reporting weekly doses in the range 0.051 to 0.147 g IgG per kg body weight [15]. Note that even for comparable body masses, due to the variability in IgG pharmacokinetics, the required IgG dosage always depends on the individual patient. Nevertheless, our simulations have a firm quantitative basis and reproduce values that are in agreement with the range of values obtained from extended experimental studies. This remains true if a constant background of bacteria is taken into account and will be discussed below.

The numerical results of our simulations are confirmed by the analytical solution of the model as derived in the Methods Section [cf Eq. (14)]. It can be rigorously calculated that the serum IgG concentration between two subsequent treatments, 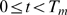, is given by:
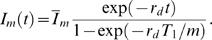
(56)In order to compare different substitution therapies, we measure the trough level of the serum IgG concentration at 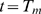:
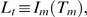
(57)imposing the condition that each treatment maintains the same trough level 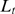. For the IgG dose per treatment 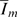, this implies the central relation
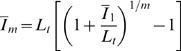
(58)in terms of the IgG dose 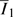. This formula reproduces the aforementioned values obtained from the simulations and, moreover, can be used to express characteristic quantities in the comparison of substitution therapies 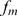 and 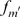. For example, the relative difference in the IgG doses per treatment is given by
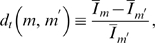
(59)and the relative difference in the time-integrated IgG doses is given by
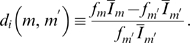
(60)


We plot 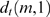 and 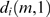 in Figure 2B and observe that the amount of substituted IgG is reduced in therapies with higher frequency 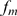, both per treatment and also time-integrated. For example, under substitution therapy with frequency 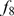 the time-integrated dose is reduced by 23% as compared to 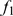. Per treatment the IgG dose is even reduced by more than 90% for substitution therapy with frequency 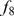 as compared to 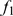. A lower IgG dose per treatment has direct consequences for the IgG peak level
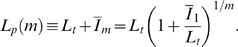
(61)This exponentially decreasing function of 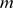 is plotted in Figure 2C. The comparison of substitution therapy 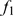 versus 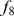 reveals a significant difference in the increase relative to the physiological trough level 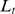 of 75% versus 7%, respectively. In absolute numbers this corresponds to 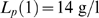 and 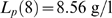 and is in agreement with the range of values found in experiment [13], [15]. During the time interval 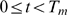 the serum IgG concentration drops from 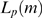 to 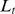, where the corresponding time-averaged serum IgG concentration is defined by
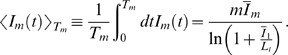
(62)As can be seen in Figure 2C, the relative deviation of 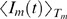 from the trough level 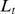 is only 3.6% for substitution therapy 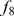, which is an order of magnitude smaller than for substitution therapy 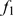 with 34%.

Next, we consider the case where fluctuations in the administered IgG dose per treatment are present. As can be observed in Figure 2D for all substitution therapies with the same fluctuation strength 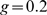, the serum IgG trough level 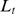 is still maintained on average. However, variations of 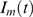 around 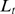 are larger for substitution therapies with lower frequency 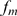. This observation can be quantified by the analytical solution of the model as presented in the Methods Section [cf Eq. (17)]. We find that the serum IgG concentration between two subsequent treatments varies around the time-dependent mean value Eq. (56) with the relative variance
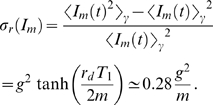
(63)Here, we used that 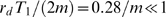 to approximate the tangens hyperbolicus in the last step. Thus, for a fixed fluctuation strength 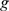 of the administered IgG dose 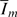, the impact on the variation of 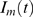 decreases as 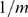 for substitution therapies with larger frequencies 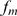. This is a consequence of the fact that in substitution therapies with lower frequency, a single fluctuation determines the time-evolution of 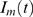 over a longer period of time. The implications of fluctuations in the presence of infections are further analyzed below.

### Substitution Therapies in the Presence of Infections

We investigate the conditions for which infections under IgG substitution therapy are either cleared or develop into chronic infections with a finite concentration of bacteria surviving the immune response. In all simulations the bacteria population is initially absent until the time point of infection at 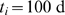. Then, starting from this time point, a finite bacteria population is proliferating and triggering the immune response. We choose the parameter values 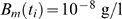 and 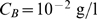, implying for a typical cell weight of 10^−9^ g a starting value of about 10^2^ bacterial cells that can grow to a population of roughly 10^8^ bacterial cells. With this in mind, in the simulations we set 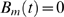 whenever 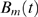 attains values below 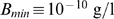. The bacteria proliferation rate is chosen to be 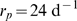 and the clearance rate 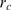 is varied.

#### Neglect of fluctuations in the administered IgG dose

**Figure 3:**
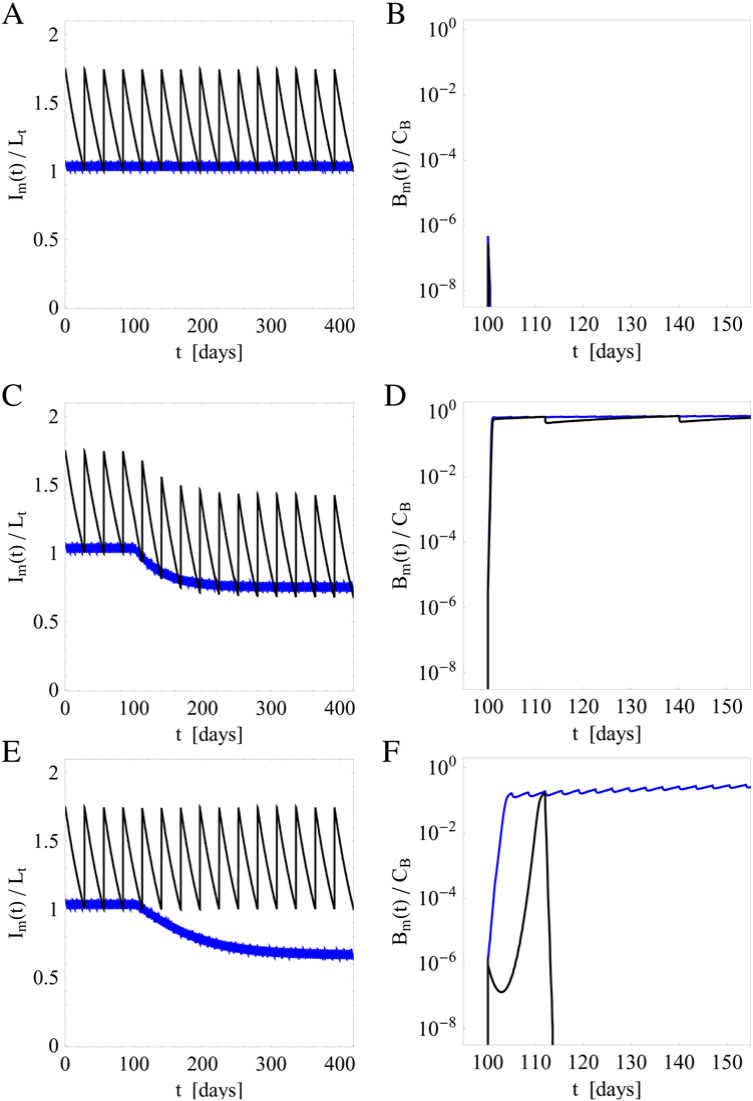
Simulation results of the immune response to infection for different clearance rates 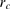 in the absence of fluctuations in the administered IgG dose per treatment. The serum IgG concentration 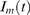 and the bacteria population 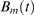 are plotted as a function of time and for substitution therapies with frequencies 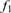 (black line) and 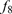 (blue line). A and B: Limit of high clearance rate with 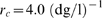. Within one day the infection is cleared by extinction of the growing bacteria population under both substitution therapies. C and D: Limit of low clearance rate with 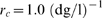. A chronic infection rapidly develops under both substitution therapies which is accompanied by a reduced serum IgG trough level. E and F: In the intermediate regime of clearance rates with 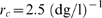 a qualitative difference is observed between substitution therapies with frequencies 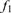 and 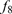. The former succeeds in clearing the infection within 14 days time, whereas the latter can not hold the bacteria population at bay such that a chronic infection develops.

In the absence of fluctuations (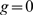), we compare the immune response under different substitution therapies. Typical simulation results are plotted in Figure 3 for substitution therapy with frequency 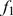 (black line) and 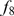 (blue line). Choosing a high value for the clearance rate, 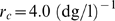, the proliferation of bacteria is strongly suppressed under both substitution therapies resulting into the fast extinction of the bacteria population and a virtually unaffected dynamics of the serum IgG concentration (Figures 3A and 3B).

The situation is different for low clearance rates, where a chronic infection develops that is accompanied by a decrease in the serum IgG concentration. This is shown in Figures 3C and 3D for the clearance rate 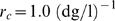 and is qualitatively similar under both substitution therapies. Due to the immune response the growth of the bacteria population is limited to a size lower than the carrying capacity 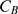. Note that the concentration 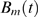 is modulated by the repeated IgG infusions and has an average value that is slightly larger for substitution therapy 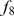 as compared to 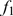. The serum IgG trough level for substitution therapy 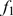 is slightly more reduced than for substitution therapy 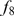. This is a general feature which can be explained in the limit of a constant bacteria population 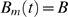 with 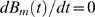. The trough level in the presence of a constant bacteria population follows directly from Eqs. (56) and (57):
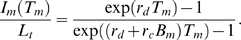
(64)For small concentrations 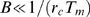, we obtain to lowest order in the size of the bacteria population:
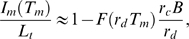
(65)where we defined
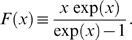
(66)This is a strictly monotonically increasing function of 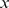. Thus, as can be expected, a small constant background of bacteria results only into a modest decrease of the serum IgG trough level. However, as follows from the functional dependence of 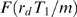 on 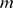, the decrease of the trough level is less pronounced for substitution therapies with higher frequency 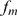. This result is preserved in the limit of a strong chronic infection, 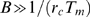, where Eq. (64) can be approximated by
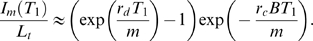
(67)In this limit, the immune response against infections consumes significantly more IgG than is lost by its natural degradation. Therefore, the serum IgG trough level becomes exponentially suppressed indicating that the immune response fails to clear the infection.

Interestingly, qualitative differences for the two substitution therapies 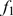 and 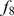 are observed at intermediate values of the clearance rate 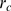. In Figures 3E and 3F we plot the result for 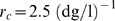 and find that a chronic infection develops for substitution therapy 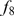 accompanied by a decrease in the corresponding serum IgG trough level. In contrast, for substitution therapy 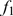 the initially increasing bacteria population is eventually cleared by the immune response. Shortly after the IgG infusion treatment at day 112 the infection is cleared, even though until this time the bacteria population had grown to a size that is comparable to the final population size of the chronic infection under substitution therapy 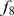.

**Figure 4:**
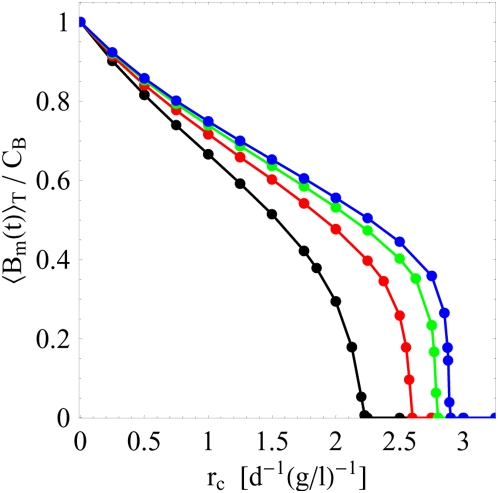
Simulation results for the time-averaged size of the bacteria population in the equilibrated system. The time-averaged size of the bacteria population, 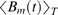, is computed as a function of the clearance rate 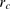 according to Eq. (68). The proliferation rate 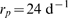 and the carrying capacity 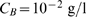 are kept fixed in the computations for different substitution therapies with frequencies 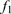 (black line), 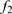 (red line), 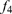 (green line), and 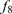 (blue line). It is clearly observed that the critical value of the clearance rate with 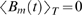 depends on the treatment frequency.

We investigate the transition from a cleared infection to a chronic infection as a function of the clearance rate 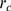. This is done by computing the time-averaged value
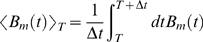
(68)in the time interval 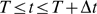. The parameters of the time interval are chosen such that the system has time to equilibrate, i.e. we start at time point 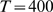 days and consider a time interval of 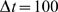 days. For different substitution therapies the results are shown in Figure 4 as a function of 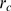. We find that the critical clearance rate above which chronic infections do not develop depends on the frequency 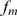 of the applied substitution therapy. Higher frequencies 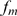 require higher values of 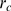 for the clearance of the infection.

The immune response model for infections under IgG substitution therapy can be solved analytically. After a sufficiently long time the system has equilibrated in response to the infection. Modulations of 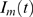 on time scales smaller than 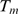 are averaged out by replacing 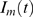 in Eq. (4) with the time-averaged serum IgG concentration 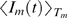. This renders the solution of the differential equation (4) a trivial task. The relevant parameter, which decides about the fate of the bacteria population due to the immune response, is given by the ratio of the bacteria proliferation rate to the clearance rate,
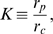
(69)and does not depend on the carrying capacity 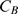.

In the limit of a sufficiently strong immune response with 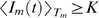, bacteria are cleared faster than they can proliferate resulting into the complete extinction of the population. In this case the decay of the bacteria population is given by
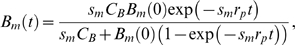
(70)where we introduced the non-negative parameter
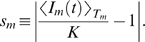
(71)This parameter depends on the applied substitution therapy and determines how fast the bacteria population becomes extinct. For finite values of 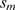, the time at which the infection is cleared, 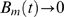, scales as 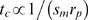.

In the limit of a weak immune response, 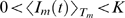, the bacteria population attains a finite size indicating the development of a chronic infection. The solution of Eq. (4) is given by
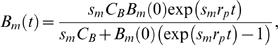
(72)such that 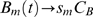 after sufficiently long times 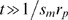. Thus, a weak immune response has two effects on the bacteria population: (i) It gives rise to a retardation of the population growth by the scaling factor 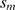, and (ii) it ultimately results into a chronic infection, where the size of the bacteria population, 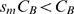, is smaller than in the absence of immune responses.

**Figure 5:**
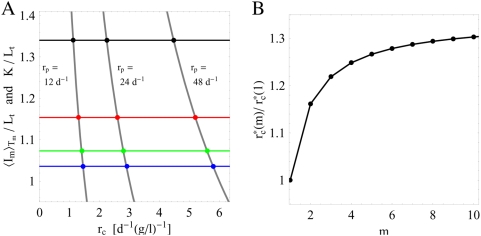
Analytical results for the critical clearance rates in the absence of fluctuations. A: The intersections between 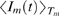 (horizontal lines) and 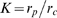 (grey lines) indicate the critical clearance rates (crossing points) according to Eq. (73). This is shown for different values of 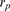 and substitution therapies with frequencies 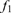 (black line), 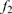 (red line), 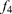 (green line), and 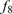 (blue line). B: Comparison of substitution therapies with different treatment frequencies 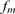 by the ratio 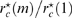 according to Eq. (74).

The transition from a system with a cleared infection to a system with a chronic infection is determined from 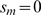, or
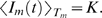
(73)In Figure 5A we plot 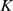 as a function of 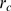 for three different values of the proliferation rate 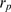. The crossing points of 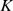 with 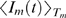 for the four substitution therapies with frequencies 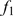, 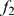, 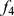, and 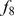 are indicated for different values of the proliferation rate 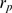. There is quantitative agreement between the values obtained from the simulations (Figure 4) and the calculated values for 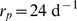. The crossing points define the critical clearance rate 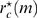 and using Eqs. (62), (69), and (73) we obtain the analytical expression:
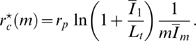
(74)The dependence of the critical clearance rate on the bacteria proliferation rate 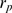 is observed in Figure 5A, implying that the success of clearing an infection under substitution therapy with frequency 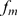 strongly depends on the virulency of the bacteria. Note, however, that the critical clearance rate does not depend on the carrying capacity 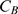.

In Figure 5B we plot the ratio 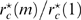 to illustrate the difference between substitution therapies with frequencies 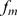. In particular, we find that the clearance of infection under substitution therapy with frequency 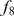 requires the critical clearance rate to be about 30% larger as compared to substitution therapy with frequency 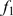 under otherwise identical conditions. This quantitative analysis explains the qualitatively different results of the simulations for the serum IgG concentration and the bacteria population presented in Figure 3. For 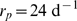, we obtain from Eq. (74) that 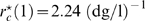 and 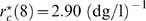. The numerical calculations in Figure 3A–3B and Figure 3C–3D are performed for parameters 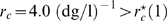, 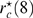 and 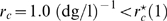, 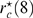, respectively, and give rise to qualitatively comparable results under both substitution therapies. In contrast, the numerical calculation in Figure 3E–3F is performed for the clearance rate 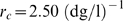 with 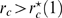 but 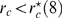, which explains the success of substitution therapy 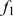 and the failure of substitution therapy 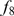 to clear the bacterial infection.

#### Impact of fluctuations in the administered IgG dose

**Figure 6:**
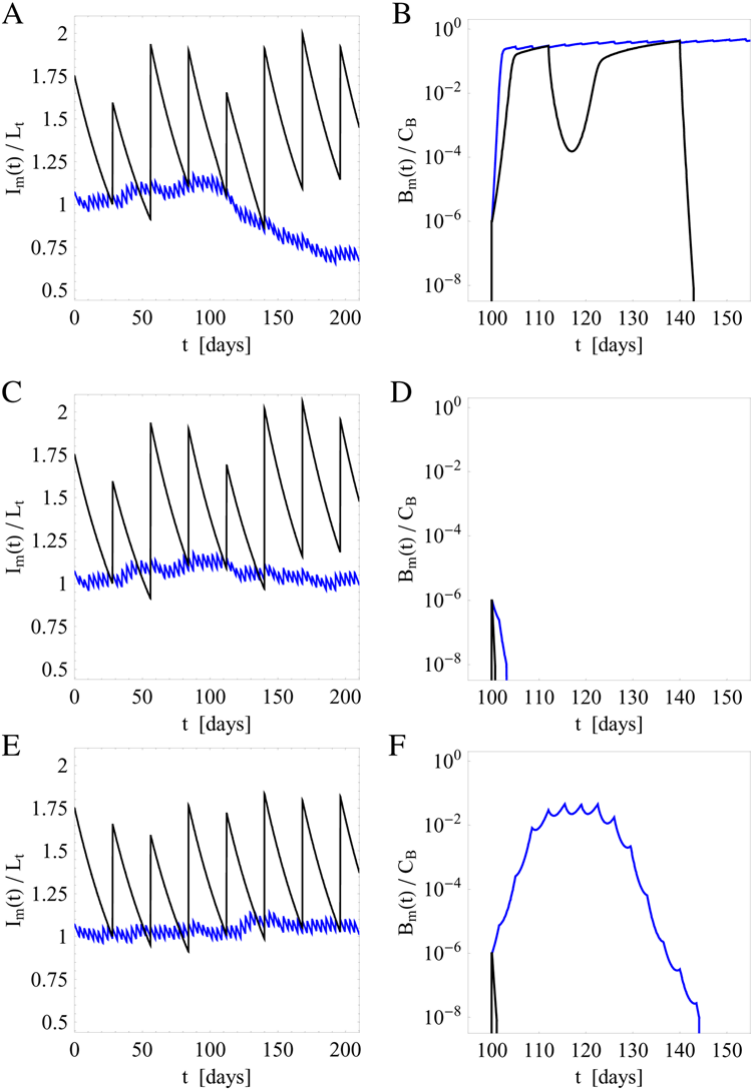
Simulation results of immune responses to infections for different clearance rates 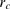 in the presence of fluctuations in the administered IgG dose per treatment with fluctuation strength 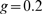. The serum IgG concentration 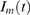 and the bacteria population 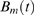 are plotted as function of time and for substitution therapies with frequencies 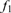 (black line) and 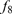 (blue line). A and B: For clearance rate 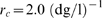 the infection is cleared after 43 days under substitution therapy with frequency 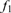, whereas a chronic infection develops under substitution therapy with frequency 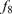. C and D: For clearance rate 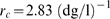 the infection is cleared within 1 and 3 days under substitution therapy with frequency 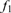 and 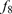, respectively. E and F: For the same parameters as in C and D but for a different random realization of fluctuations in the administered IgG dose per treatment. Even though the infection is ultimately cleared, this is only achieved after 44 days under substitution therapy with frequency 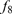.

In the presence of fluctuations, we compare the immune response under substitution therapies with frequencies 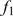 and 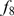. Typical simulation results are plotted in Figure 6. These are obtained for the fluctuation strength 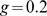 and for clearance rates 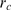 that would induce a chronic infection in the absence of fluctuations. As can be deduced from Figure 4, a finite bacteria population size of about 30% of the carrying capacity 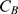 implies that 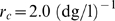 for substitution therapy with frequency 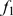 and 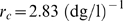 for 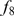.

In Figures 6A and 6B, we plot 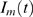 and 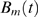 for 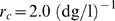 and for substitution therapies with frequencies 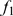 (black line) and 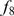 (blue line). While in the absence of fluctuations a chronic infection would develop under both substitution therapies, in the presence of fluctuations this is only observed for substitution therapy with frequency 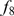. The substitution therapy with frequency 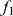 gives rise to the extinction of the bacteria population, where the infection is cleared 43 days after the time point of infection at 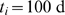. During this time period the bacteria population size is fluctuating over many orders of magnitude and even reaches values close to 30% of 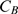 but is ultimately cleared. Note that under substitution therapy with frequency 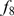 the bacteria population grows to 56% of 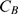, which corresponds to the size expected in the absence of fluctuations (Figure 4).

Increasing the clearance rate to 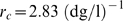, we plot in Figures 6C and 6D the corresponding simulation results for 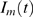 and 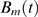. For both substitution therapies the extinction of the bacteria population is observed, where the infection is cleared within 1 day for substitution therapy with frequency 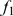 and within 3 days for 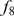. However, we stress that different random realizations in the administered IgG dose per treatment can give rise to very different courses of the infection. For example, in Figures 6E and 6F we show the results for parameter values that are identical to the ones used in Figures 6C and 6D but for a different random realization in the administered IgG dose per treatment. In this case it takes about 44 days before the infection is cleared by substitution therapy with frequency 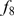. In general, different random realizations give rise to large variations in the infection duration that can exceed months and years. Therefore, even though the infection may be ultimately cleared, it may still have to be treated as a chronic infection in the sense that additional medication is necessary to clear the infection on a reasonable time scale. This issue will be further addressed below.

The simulations suggest that the critical clearance rate in the presence of fluctuations, 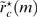, is reduced compared to the case of absent fluctuations: 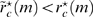. Conversely, it can be argued that for the same clearance rate infections are cleared by time-averaged serum IgG concentrations in the presence of fluctuations, 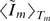, that are lower compared to 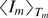 in the absence of fluctuations. The reason for this being that in the course of time fluctuations can be both to the disadvantage and to the advantage of infection clearance. However, once an infection has been cleared, the bacteria population has died out and is defeated for ever. As is observed in Figure 6, infections are in fact cleared by victorious fluctuation that happen to occur in the course of time. For example, analyzing substitution therapy with frequency 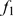 in Figure 6A, the current peak level at the time point of infection is relatively low and is only increasing above average values in the subsequent treatment at day 140, which then leads to the immediate clearance of the infection. For substitution therapy with frequency 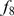 in Figure 6C, such a victorious fluctuation happens to occur around the time point of infection resulting into the immediate clearance of the infection, whereas in Figure 6E it takes until day 125 before a victorious fluctuation is established that ultimately defeats the infection.

**Figure 7:**
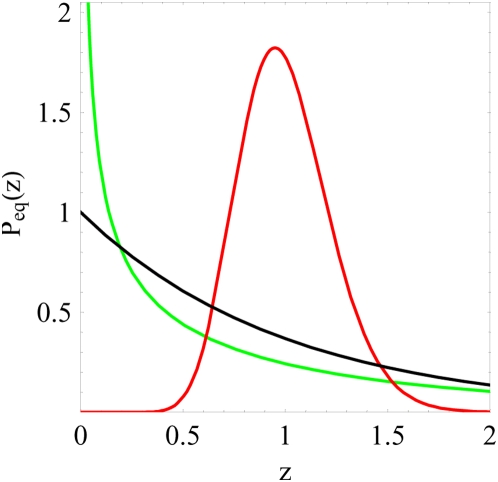
Qualitative change in the equilibrium probability distribution 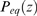. The equilibrium probability distribution Eq. (75) is plotted for different values of the parameter 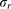. For 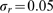 (red line) the distribution vanishes, 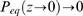, indicating that the infection is not cleared. For 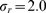 (green line) the distribution diverges, 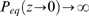, making the clearance of infection the most likely event. The transition occurs for 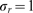 (black line) where 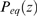 equals the exponential distribution.

Analytical results are obtained by translating the stochastic differential equation (4) into the corresponding Fokker-Planck equation [28]. The latter describes the time-evolution of the probability distribution 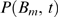 for the time-dependent survival of a bacteria population under the immune response. The derivation of the stationary probability distribution 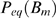 for the equilibrated system is presented in the Methods Section [cf Eq. (44)]. Here, we readily give the final expression in a compact form:
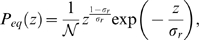
(75)where the normalization constant 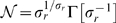 contains the Gamma-function 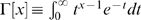. Furthermore, we use the scaled variable
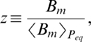
(76)while the parameter of the distribution corresponds to the relative variance
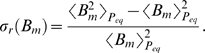
(77)Interestingly, two distinct regimes of 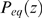 are identified that are visualized in Figure 7. For 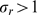 the probability distribution Eq. (75) diverges at 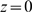, such that the extinction of the bacteria population is most likely to occur in this regime. In contrast, for 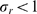 we find that 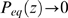 for 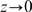, meaning that in this regime the extinction of the bacteria population is impossible and a chronic infection is established. The abrupt transition between these two regimes is induced by the fluctuations and occurs at 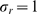, where 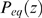 equals the exponential distribution with a finite value at 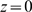.

**Figure 8:**
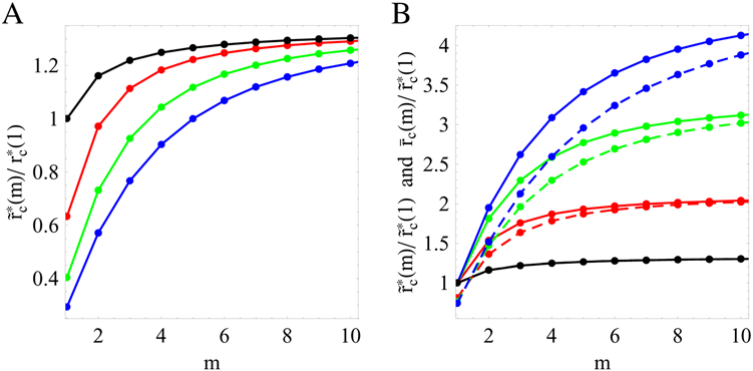
Analytical results for the critical clearance rates in the presence of fluctuations. A: The ratio 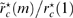 [cf Eq. (80)] reveals that the critical clearance rate decreases with increasing fluctuation strength 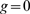 (black line), 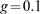 (red line), 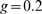 (green line), and 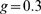 (blue line). B: For clearance rates above 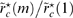 (solid lines) the infection is cleared [cf Eq. (80)], whereas a chronic infection develops for clearance rates below 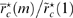 (dashed lines) [cf Eq. (84)]. The dependence of the critical clearance rate on the applied substitution therapy becomes increasingly significant for increasing fluctuation strength 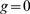 (black line), 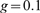 (red line), 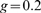 (green line), and 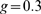 (blue line).

The average size of the bacteria population has been calculated in the Methods Section [cf Eq. (41)] and yields in terms of the model parameters:
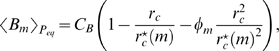
(78)where the non-negative quantity
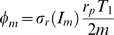
(79)contains the relative variance Eq. (63). Note that, due to the fluctuations, the bacteria population vanishes already at lower values of the clearance rate as compared to 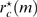 in the absence of fluctuations (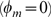). The critical clearance rate in the presence of fluctuations is calculated from the condition 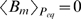 and is given by:
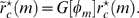
(80)Here, we defined the strictly monotonic function
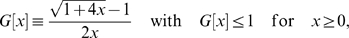
(81)such that 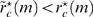 in the presence of fluctuations. Note that, since 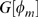 increases with larger values of 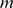, the effect of fluctuations is more pronounced for substitution therapies with lower frequencies 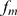. This can be seen in Figure 8A, where we plot the ratio 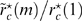 as a function of 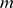 and for different values of the fluctuation strength 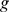. For a given fluctuation strength, infections are cleared for clearance rates above the solid line. There is quantitative agreement between the clearance rates used in the simulations with fluctuation strength 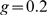 (Figure 6) and the values obtained from the analytical calculations (Figure 8A). For example, for substitution therapy with frequency 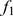 we used 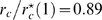 in the simulations. This value is below 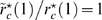 for 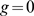 but above 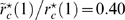 for 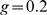. Correspondingly, for substitution therapy with frequency 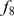 we used 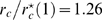, which is below 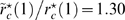 for 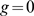 but above 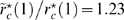 for 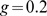.

In order to compare substitution therapies in the presence of fluctuations among each other, we plot the ratio 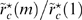 in Figure 8B as solid lines. It is important to notice that, in comparison to the case of absent fluctuations, the difference between substitution therapies in the presence of fluctuations is significantly larger. For 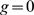 we found that the clearance of infection under substitution therapy with frequency 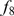 implies that the critical clearance rate is about 30% larger as compared to substitution therapy with frequency 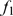 (Figure 5B). However, for fluctuation strength 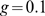, 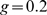, and 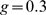 the critical clearance rate under substitution therapy with frequency 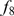 is increased by, respectively, 100%, 200%, and 300% relative to the substitution therapy with frequency 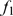.

Next, as is shown in the Methods Section [cf Eq. (42)], the calculation of the average square of the bacteria population concentration yields
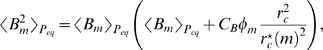
(82)such that the relative variance becomes
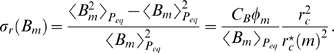
(83)The variance diverges for 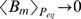 indicating the transition from a chronic infection to the extinction of the bacteria population. The extinction of the bacteria population is impossible for 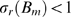 and the clearance rate that separates the two regimes follows from the condition 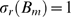, or:
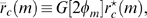
(84)with 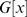 as defined in Eq. (81). In Figure 8B, the ratio 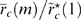 is plotted as dashed lines for the different fluctuation strengths 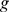. The model predicts that for clearance rates below the dashed lines a chronic infection develops. For clearance rates above the solid lines the bacteria population becomes extinct by the immune response. In the narrow regime between the dashed and solid lines the bacteria population may either become extinct or attain a finite size.

**Figure 9:**
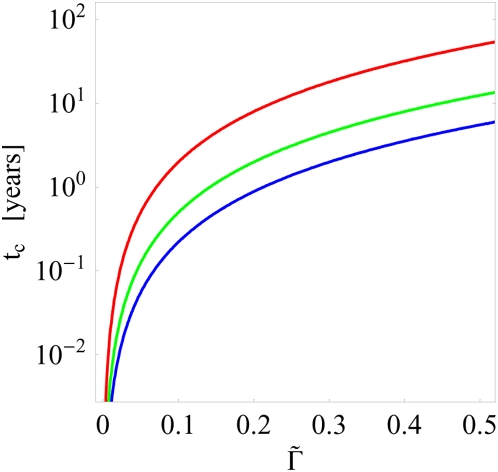
Infection clearance time as a function of shape space inhomogeneities for different values of the fluctuation strength. The clearance time 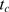 [cf Eq. (87)] as a function of 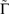 [cf Eq. (86)] for different fluctuation strengths 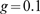 (red line), 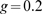 (green line), and 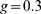 (blue line). For 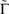 larger than a few percent, the duration of infection rapidly exceeds months and years.

The derivation of the equilibrium probability distribution Eq. (75) entails that all time-dependent information is lost. As has been stated above, an infection might be cleared at some day but the duration of infection may exceed months and years. We estimate the clearance time in the presence of fluctuations that compensate for a reduced time-averaged IgG concentration: 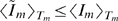. The condition Eq. (73), which was derived in the absence of fluctuations, translates in formal analogy to Eq. (7) into
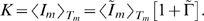
(85)From the viewpoint of the bacteria population, the non-negative factor
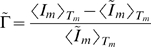
(86)can be interpreted as a measure for inhomogeneities in the IgG shape space distribution. In order to clear the infection, the deficit 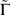 has to be compensated by victorious fluctuations in the course of time. The time for such a victorious fluctuation to emerge during 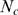 infusion treatments defines the clearance time: 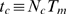. It is shown in the Methods Section that 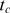 can be calculated by averaging the sum of fluctuations for 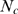 subsequent treatments under the constraint that this sum compensates for the deficit 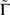. We obtain the resulting expression [cf Eq. (55)]:
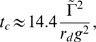
(87)which is independent of the treatment frequency. In Figure 9 we plot 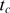 for different values of the fluctuation strength 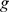 in the range 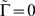 (or: 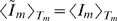) to 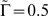 (or: 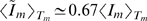). A clearance time in the order of days is only obtained for 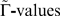 of a few percent. For 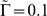, or 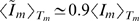, the estimated clearance time already amounts to about 80 days, 6 months, and 2 years for fluctuation strengths 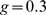, 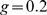, and 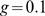, respectively. Thus, in the presence of fluctuations infections are cleared that would otherwise become chronic, however, since the time required for a victorious fluctuation to emerge can exceed months, fluctuations do not represent a failsafe mechanism of infection clearance.

## Discussion

Applying a stochastic immune response model, we perform a comparative study of IgG substitution therapies. The combined analysis of computer simulations and analytical calculations permits to make quantitative predictions that are of therapeutic relevance. We base our model on data that are obtained from large-scale studies of IgG substitution therapies [15]. However, to our knowledge, clinical studies where serum IgG levels of a sufficiently large group of patients are monitored for reasonably long times under IgG substitution therapy with varying treatment frequencies do not yet exist. This may be related to the fact that XLA, with an estimated number of 1500 new patients per year worldwide, is a relatively rare immune deficiency. Therefore, mathematical modeling represents the ideal tool for simulating and optimizing IgG substitution therapies under uniquely defined conditions. As a general result of this approach, the behavior of selected quantities is predicted and motivates purposeful experiments.

For IgG substitution therapies with different treatment frequencies we compare serum peak levels and IgG dosages that are required to maintain the same serum trough level. In general, we find that the amount of substituted IgG is reduced for substitution therapies with higher treatment frequencies, both per treatment and also time-integrated (Figure 2B). Thus, against the background of the current worldwide IgG shortage and the continuously increasing high costs in the order of 100 US $ per gram IgG [20], [21], substitution therapies with high treatment frequencies are preferable. In particular, for substitution therapy with two treatments per week relative to substitution therapy with one treatment per four weeks, we find that the IgG dose per treatment is reduced by more than 90% and the time-integrated dose is reduced by more than 20%. This has direct consequences for the serum IgG peak level and the time-averaged serum IgG concentration. Both quantities are exponentially decreasing with the frequency of the substitution therapy (Figure 2C). Comparing again substitution therapies with two treatments per week and with one treatment per four weeks, we obtain a significant difference in the increase of the serum IgG peak level relative to the physiological trough level of 7% versus 75%, respectively. Similarly, we find an order of magnitude difference in the deviation of the time-averaged serum IgG concentration from the trough level, which is only 3.6% for substitution therapy with two treatments per week as compared to 34% for substitution therapy with one treatment per four weeks. In general, it can be deduced from Figure 2B and 2C that the characteristic quantities quickly level off for treatment frequencies above once per two weeks.

All these findings support IgG substitution therapies with high treatment frequencies for which the IgG consumption is reduced and where therapeutic serum IgG levels are kept close to physiological levels. This is highly desirable since the precise mechanisms behind immunomodulatory effects of administered IgG are not yet understood, however, it is generally accepted that administered IgG does interfere with the immune system at many different levels [14], [31], [32]. For example, administered IgG has inhibitory effects on antigen presentation [33], on T cell activation [32], [34], [35], on cellular cross-talk via the cytokine network [36], [37], and on phagocytosis via the IgG fragment crystallizable (Fc) region [32], [38], [39]. In general, impaired immune regulation by Fc receptors leads to unresponsiveness or hyperreactivity to non-self as well as self antigens [40]. Moreover, it is reported that administered IgG promotes apoptosis in lymphocytes and monocytes [32], [41]. Taken together, there is sufficient reason for keeping serum IgG levels close to the physiological level.

It should be kept in mind, however, that the primary goal of IgG substitution therapy in XLA patients is the prevention of opportunistic infections. At this point we exploit the advantage of mathematical modeling where different substitution therapies in the presence of bacterial infections can be analyzed under identical conditions as a function of the clearance rate. In general, the value of the clearance rate, which depends on microscopic details of the binding between IgG and antigenic epitopes [42], is not known. We vary this parameter and find that the infection is cleared under any IgG substitution therapy for sufficiently high clearance rates. However, a critical value of the clearance rate exists below which a chronic infection develops that is accompanied with a decrease in the IgG trough level. As can be seen in Figure 3C and Figure 3D, the bacteria population survives the immune response and grows by a factor 10^6^, while the IgG trough level is only lowered by a factor 0.75. We conclude that small changes in the trough level can be associated with large changes in the bacteria population. Therefore, monitoring the serum IgG trough level in XLA patients on a regular basis provides information for the early detection of infections by systematic deviations.

Our analytical calculations reveal that the critical clearance rate strongly depends on the virulency of the bacteria, since it is proportional to the bacteria proliferation rate. Most importantly, however, the critical clearance rate depends on the applied substitution therapy, as we consistently show by numerical (Figure 4) and analytical (Figure 5) calculations. In general, a higher critical clearance rate is required for substitution therapies with higher treatment frequencies. This supports IgG substitution therapies with low treatment frequencies in order to optimize the prevention of chronic infections. For example, the substitution therapy with two treatments per week requires the critical clearance rate to be about 30% larger as compared to substitution therapy with one treatment per four weeks (Figure 5B). The difference between substitution therapies becomes even more significant in the presence of fluctuations in the administered IgG dose. Comparing the same substitution therapies as before, the predicted increase in the critical clearance rate is of the order of several hundreds of percent with the exact value depending on the fluctuation strength (Figure 8). Therefore, ignoring the potential impact of immunomodulatory effects and the important issue of IgG shortage, treatment frequencies well below once per week are preferred.

The differences in the critical clearance rate for different substitution therapies are minimized by keeping the fluctuations in the administered IgG dose per treatment small. From the viewpoint of the considered bacteria species, these fluctuations can also be interpreted to effectively model dynamic inhomogeneities of the IgG shape space distribution during the immune response (Figure 1B). Even if the time-averaged serum IgG concentration in the relevant shape space area is smaller than is required for the clearance of the infection, it may nevertheless be cleared in the course of time due to victorious fluctuations. Thus, in principle, due to the presence of fluctuations infections are cleared that would otherwise become chronic. However, our model predicts that only small inhomogeneities of the order of several percent are balanced by victorious fluctuations on a reasonable time scale. For larger inhomogeneities the time required for clearing the infection can easily exceed months (Figure 9), making additional medication necessary to clear the infection on a reasonable time scale. A side effect of fluctuations is that systematic deviations in the monitored serum IgG trough level get blurred (Figure 6).

In conclusion, the stochastic immune response model emphasizes the importance of elaborate IgG pooling in order to achieve a highly homogeneous IgG shape space distribution for the reliable clearance of infections on a reasonable time scale. For XLA patients the choice of the treatment frequency is a trade-off between competing interests: On the one hand, therapeutic IgG levels should be kept close to physiological levels in order to diminish immunomodulatory effects and to make good economic sense. This is realized by substitution therapies with high treatment frequencies. On the other hand, the regime of clearance rates that are effective at clearing infections is to be maximized. This is achieved by substitution therapies with low treatment frequencies. Taken together, our model suggests that the compromise solution with regard to the treatment frequency of IgG substitution therapy for XLA patients ranges from once per week to once per two weeks.

We finally note that the stochastic immune response model is a first step that can be improved in various ways. For example, the time-evolution of the serum IgG concentration could be more realistically represented in the shape space of IgG specificities to model the simultaneous infection by different bacteria species explicitly. Furthermore, the impact of immunomodulatory effects due to high serum IgG peak levels could be included into the model and analyzed by a comparative study of substitution therapies. Leaving these and related issues for future research, the present study already represents a conclusive example for the potential of mathematical modeling in optimizing empirical IgG treatment protocols. On the basis of our analysis, we suggest clinical studies where the same group of XLA patients is monitored during sufficiently long times for infections and serum IgG levels under different IgG substitution therapies within the specified range of optimal treatment frequencies.
